# From Benign Symptoms to Subarachnoid Hemorrhage: A Pediatric Case

**DOI:** 10.7759/cureus.104188

**Published:** 2026-02-24

**Authors:** Hayian H Omran, Abdulrahman H AlQaderi, Maitha Almazrouei

**Affiliations:** 1 Internal Medicine, RAK Medical and Health Sciences University, Ras Al Khaimah, ARE; 2 Psychiatry, Emirates Health Services (EHS), Dubai, ARE; 3 Emergency Medicine, Sheikh Khalifa General Hospital, Umm Al Quwain, ARE

**Keywords:** diagnostic delay, neuroimaging, pediatric intracranial aneurysm, posterior communicating artery aneurysm, subarachnoid hemorrhage

## Abstract

Pediatric intracranial aneurysms are rare and represent an uncommon but life-threatening cause of subarachnoid hemorrhage in children. Their diagnosis is usually delayed because early symptoms are nonspecific and often mimic benign pediatric conditions. We report the case of a 13-year-old boy who presented with a one-week history of progressive headache and vomiting and was repeatedly evaluated at multiple healthcare facilities, where he was treated for suspected gastroenteritis and sinusitis. He clinically declined on the day of presentation with sudden worsening of headache, photophobia, and an episode of generalized tonic-clonic seizure, prompting emergency transfer to our institution. He was fully oriented on examination with no focal neurological deficits, but was drowsy, with neck stiffness. Non-contrast computed tomography of the brain demonstrated diffuse subarachnoid hemorrhage with intraventricular extension and mild hydrocephalus. Computed tomography angiography subsequently identified a ruptured aneurysm arising from the right posterior communicating artery as the source of the bleed. The patient was managed with close neurological monitoring, seizure prophylaxis, and measures aimed at controlling intracranial pressure. As neurointerventional services were not available locally, arrangements were made for urgent transfer to a tertiary neurovascular center. At the tertiary center, an external ventricular drain was placed for intracranial pressure control. The patient subsequently made a good neurological recovery and was discharged without residual deficits. This case highlights the diagnostic challenges of aneurysmal subarachnoid hemorrhage in children and emphasizes the importance of early neuroimaging in patients with persistent or progressive neurological symptoms.

## Introduction

Pediatric intracranial aneurysms are considered to be uncommon, accounting for only 1-5% of intracranial aneurysms, and are a small subset of cerebrovascular diseases in children. However, they are a known cause of spontaneous subarachnoid hemorrhage. Unlike aneurysms in adults, pediatric aneurysms may have different morphologies and patterns of vascular distribution, with a relatively higher incidence of the internal carotid and posterior circulation, and lower association with traditional vascular risk factors such as hypertension and atherosclerosis. These differences may make it more difficult to diagnose in the early stages, especially when symptoms are non-specific [1,2].

Common pediatric conditions, such as headache and vomiting, are commonly associated with minor conditions like sinusitis, gastroenteritis, and viral infections. On the other hand, the presence of progressive or worsening headaches, vomiting, neck pain, or the development of neurological symptoms should alert the physician to the possibility of intracranial disease. The absence of these warning signs may lead to delayed diagnosis of aneurysmal subarachnoid hemorrhage, which may potentially carry a higher risk of morbidity [2,3].

Although posterior communicating artery aneurysms are common in adults, they are rarely seen in children. Much of the available literature consists of case series and individual case reports. When a rupture happens, bleeding can move into the ventricular system and subarachnoid space. This can cause hydrocephalus and a quick decline in neurological function, requiring urgent neurocritical care and prompt access to specialized services [1,3].

We report a 13-year-old boy with a ruptured posterior communicating artery aneurysm who initially presented with headache and vomiting and was managed on several occasions as having a benign illness before neurological deterioration occurred. The progression of symptoms in this case illustrates the difficulty of distinguishing serious intracranial pathology from common pediatric complaints and underscores the importance of early neuroimaging when symptoms persist or evolve, particularly in the emergency setting.

## Case presentation

A 13-year-old boy with no known chronic medical conditions and no prior history of seizures was brought to the emergency department by ambulance after experiencing his first generalized tonic-clonic seizure. According to his mother, the event occurred at home in the early morning hours and lasted approximately two minutes. During the seizure, the patient became unresponsive and exhibited generalized limb stiffening, teeth clenching, and eye staring, followed by a post-ictal period of drowsiness.

In the week preceding presentation, the patient had been experiencing a persistent headache, predominantly frontal in location, associated with early-morning vomiting and dizziness. Importantly, the persistence of headache for one week, early morning vomiting, and lack of response to symptomatic treatment represented clinical red flags. Although these symptoms are common in benign pediatric conditions, their progressive nature should prompt reconsideration of intracranial pathology, particularly when symptoms evolve or fail to improve. During this time, he was evaluated at multiple healthcare facilities and was initially treated for gastritis and later acute sinusitis. He received oral antibiotics and analgesics; however, his symptoms did not improve. There was no history of fever during this period.

On the day of admission, the headache suddenly worsened in intensity and extended to involve the occipital region, accompanied by neck pain. No vomiting was reported at that time. At that point, there was no history of recent head trauma. His mother reported that he had been involved in a road traffic accident approximately one month earlier, in which the vehicle overturned; however, he remained asymptomatic afterward and did not seek medical care.

Following the seizure, on arrival at the emergency department, the patient appeared sleepy but was easily arousable and able to respond appropriately to questions. His vital signs showed a blood pressure of 138/82 mmHg, heart rate of 89 beats per minute, respiratory rate of 20 breaths per minute, temperature of 36.3 °C, and oxygen saturation of 98% on room air. His weight was recorded as 36 kg. Neurological examination demonstrated neck stiffness with pain on flexion, without any focal neurological deficits. Cranial nerve examination was normal, motor strength was full in all extremities, sensation was intact to light touch, and gait was normal. The Glasgow Coma Scale score was 15/15. Examination of other systems was unremarkable, and the patient was afebrile and hemodynamically stable.

**Figure 1:**
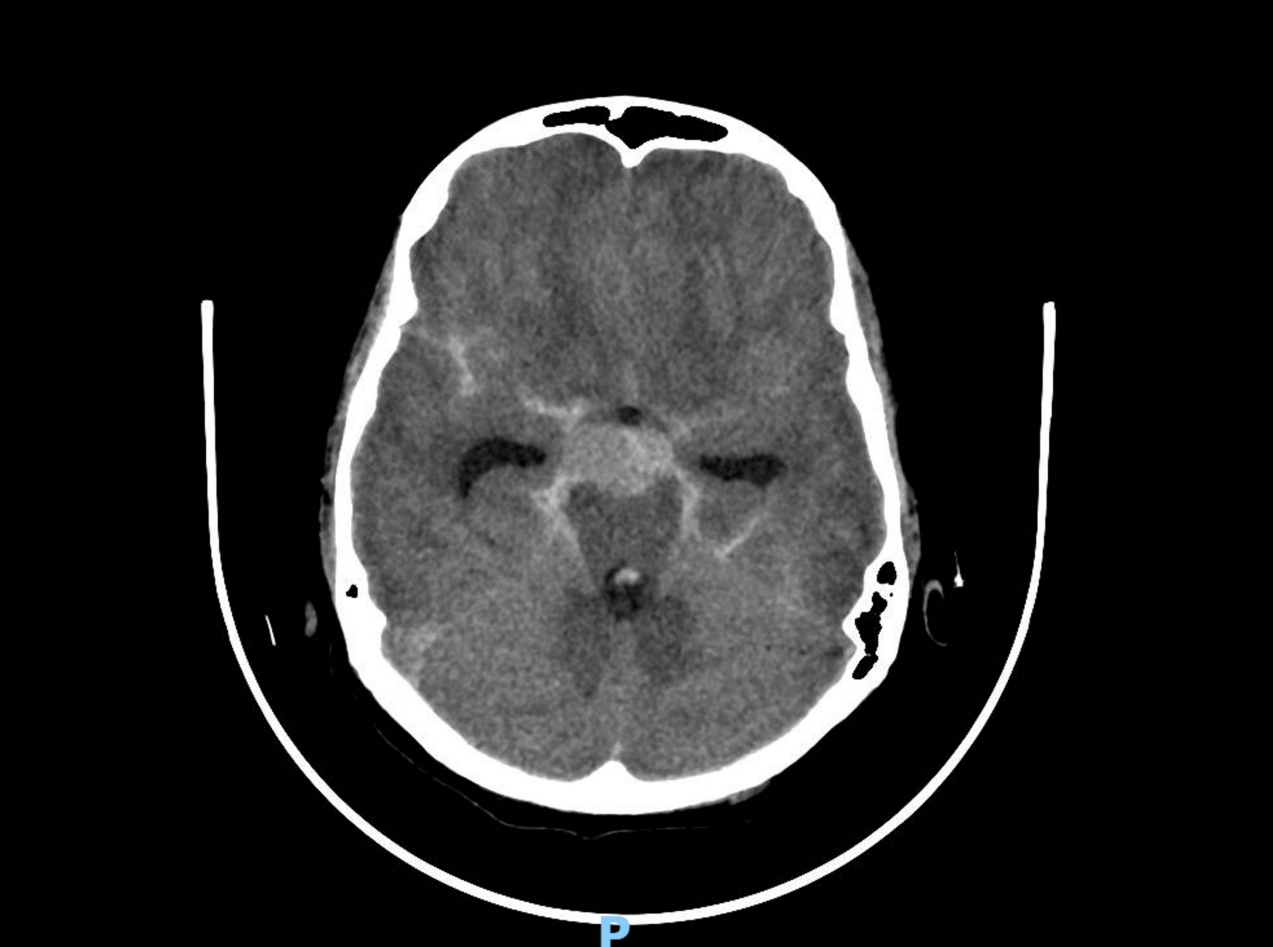
Non-contrast CT brain (axial view) demonstrating hyperdensity within the basal cisterns consistent with acute subarachnoid hemorrhage, with associated mild dilatation of the temporal horns

Initial evaluation in the emergency department included laboratory investigations, including coagulation studies (prothrombin time (PT), activated partial thromboplastin time (APTT), and international normalized ratio (INR)), which were either unremarkable or pending at the time of assessment (Table 1). Given the combination of severe headache, seizure, and signs of meningeal irritation, an urgent non-contrast computed tomography scan of the brain was obtained. Imaging demonstrated diffuse subarachnoid hemorrhage with intraventricular extension involving the lateral, third, and fourth ventricles, along with mild hydrocephalus (Figure 1). The emergency physician was informed immediately following image acquisition. Based on the clinical presentation and radiological findings, the patient was classified as Hunt and Hess grade II, consistent with moderate to severe headache and neck stiffness.

**Table 1:** Initial laboratory investigations on admission, prior to neuroimaging

Laboratory Parameter	Result	Reference Range
Complete Blood Count		
White blood cell count	19.75 ×10⁹/L	4.0–11.0 ×10⁹/L
Red blood cell count	4.88 ×10¹²/L	4.0–5.5 ×10¹²/L
Hemoglobin	12.9 g/dL	12.0–16.0 g/dL
Hematocrit	37.80%	36–46 %
Mean corpuscular volume (MCV)	77.5 fL	80–96 fL
Mean corpuscular hemoglobin (MCH)	26.4 pg	27–33 pg
Mean corpuscular hemoglobin concentration (MCHC)	341 g/L	320–360 g/L
Red cell distribution width (RDW-CV)	13.50%	11.5–14.5 %
Platelet count	353 ×10⁹/L	150–450 ×10⁹/L
Mean platelet volume (MPV)	9.7 fL	7.5–11.5 fL
Renal Function and Electrolytes		
Glucose (random)	7.5 mmol/L	3.9–7.8 mmol/L
Creatinine	49.00 µmol/L	30–70 µmol/L
Blood urea nitrogen (BUN)	2.7 mmol/L	2.5–6.4 mmol/L
Sodium	135.0 mEq/L	135–145 mEq/L
Potassium	4.0 mEq/L	3.5–5.0 mEq/L
Chloride	104 mEq/L	98–107 mEq/L
Coagulation Profile		
Prothrombin time	11.7 sec	10–14 sec
International normalized ratio (INR)	1.1	≤1.2
Activated partial thromboplastin time (aPTT)	27.3 sec	25–35 sec
D-dimer	1.95 mg/L	<0.5 mg/L
Inflammatory and Metabolic Markers		
C-reactive protein	4.8 mg/L	<5 mg/L
Lactic acid	2.4 mmol/L	0.5–2.2 mmol/L
Ammonia	<10 µmol/L	<35 µmol/L

Shortly thereafter, the patient was admitted to the intensive care unit under the care of the pediatrics and neurosurgery teams for close neurological monitoring. Initial management included elevation of the head of the bed to 30 degrees, maintenance of euvolemia with isotonic intravenous fluids, strict blood pressure monitoring with age-appropriate targets, avoidance of hypotonic solutions, and seizure prophylaxis with intravenous levetiracetam using weight-based dosing.

**Figure 2:**
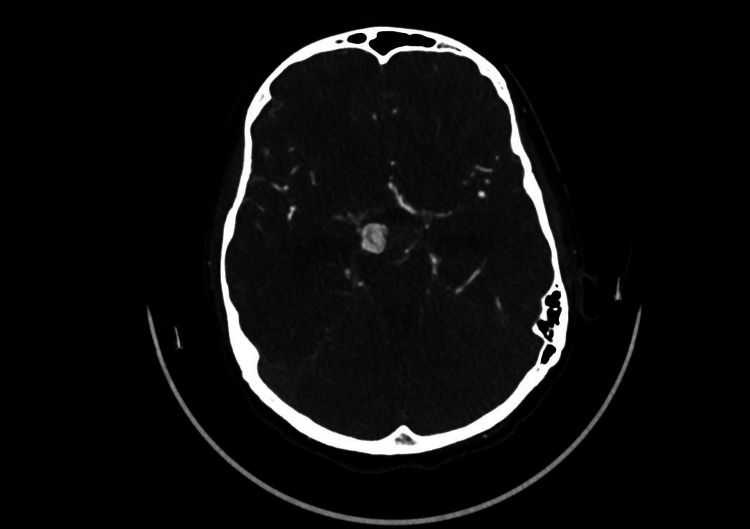
CT cerebral angiography (axial view) demonstrating a well-circumscribed saccular aneurysm arising from the right posterior communicating artery, clearly visualized within the circle of Willis

Following stabilization, computed tomography angiography of the brain and supra-aortic vessels was performed and revealed an aneurysmal dilatation arising from the posterior aspect of the right internal carotid artery, most consistent with a right posterior communicating artery aneurysm (Figure 2). No additional aneurysms, vascular malformations, or abnormalities of the vertebrobasilar circulation were identified.

During his stay in the intensive care unit, the patient remained fully conscious but continued to report severe headaches. He developed intermittent episodes of bradycardia and hypertension, raising concern for increased intracranial pressure. Following discussion with the pediatric intensive care and neurosurgery teams, hyperosmolar therapy with intravenous mannitol was initiated, with close monitoring of neurological status and serum electrolytes. Elective intubation was considered but deferred, as the patient remained neurologically stable.

Given the absence of neurointerventional services at the treating facility, urgent referral to a tertiary neurovascular center was arranged. The patient was subsequently transferred, where an external ventricular drain was placed for cerebrospinal fluid diversion and intracranial pressure control as part of definitive management. On follow-up, the patient made a favorable neurological recovery and was discharged without residual neurological deficits.

Based on the initial presentation of persistent headache and vomiting in a previously healthy adolescent, the differential diagnosis included migraine with atypical features, intracranial infection such as meningitis, sinus-related complications, intracranial mass lesion, and delayed post-traumatic pathology. However, the progression of symptoms and subsequent neuroimaging confirmed aneurysmal subarachnoid hemorrhage as the underlying etiology.

## Discussion

Epidemiology and rarity of pediatric aneurysmal subarachnoid hemorrhage (SAH) 

Pediatric aneurysmal SAH is bleeding into the subarachnoid space caused by a rupture of an intracranial aneurysm in a child or adolescent. Although SAH is well-recognized in adults, it is rare in children and represents only a small fraction of pediatric cerebrovascular events [4]. In a 30-year prospective institutional database, children (≤18 years) comprised just 47 of 4500 patients (~1.0%) with intracranial aneurysms, yet 53.2% of these children presented with SAH compared with 36.4% of adults, suggesting rupture presentations are proportionally common among affected children despite low absolute numbers [4]. Population-based data similarly underscore the etiologic role of aneurysms in pediatric SAH. In a Northern Californian cohort of 2.3 million children followed for a mean of 3.5 years, the incidence of spontaneous hemorrhagic stroke was 1.4 per 100,000 person-years, aneurysms accounted for 13% of hemorrhagic strokes overall, and among children with pure SAH, 57% had an underlying aneurysm [5]. Collectively, these findings indicate that while pediatric aneurysmal SAH is uncommon in absolute terms, aneurysms are a leading cause of spontaneous SAH in children, and pediatric aneurysm cases appear more likely than adult cases to present with hemorrhage; importantly, this rarity can reduce clinical suspicion and familiarity, contributing to delayed recognition and diagnosis.

Sentinel headaches and delayed diagnosis

In this case, the headache pattern was not classic thunderclap. Instead, it was progressive, worsening over a week, and atypical because it persisted despite treatment for presumed benign illness. That trajectory, particularly when paired with recurrent vomiting and later meningeal features, should itself have triggered early neuroimaging rather than repeated symptomatic treatment.

Pediatric experience shows how easily aneurysmal disease can be missed when presentation is atypical, and suspicion is low, even when hemorrhage is already present. This has been reported in a two-year-old child in whom subarachnoid and intraventricular hemorrhage were initially identified, yet the underlying aneurysm was not diagnosed until a later presentation, illustrating how failure to pursue vascular imaging can prolong time to definitive diagnosis in children [6].

Adult data can be used to support the general principle that SAH is still misdiagnosed when early features are mild or non-classical. In one adult cohort, 13.7% of aneurysmal SAH cases were initially misdiagnosed, often with delays exceeding 24 hours, and lower radiographic severity was associated with delayed recognition, consistent with the idea that less dramatic early presentations are easier to miss [7]. The pediatric implication is straightforward. Children may not present with a single dramatic “worst headache” moment, and clinicians should treat a progressive or evolving headache pattern as a warning signal in itself, with a deliberately low threshold for neuroimaging when symptoms persist, worsen, or change character [6,7].

Seizures as a presenting sign of aneurysmal SAH

Seizures may constitute part of the clinical presentation in pediatric patients with cerebral aneurysms and associated SAH. In a reported case of a 12‑year‑old female with a ruptured aneurysm, the patient developed generalized tonic‑clonic seizures 72 hours after the onset of a sudden, intense headache in association with subarachnoid and intraparenchymal hemorrhage [8].

The clinical spectrum of pediatric cerebral aneurysms extends beyond headache and hemorrhagic findings to include seizures among presenting signs. In a literature review of pediatric aneurysm presentations, seizures were noted as one of the possible clinical manifestations alongside severe headache and motor‑sensory deficits in children with ruptured aneurysms and hemorrhage [8].

Additionally, a broader series of pediatric intracranial aneurysms has documented seizures as a presenting symptom in affected children. In one institutional cohort, seizures were reported in approximately 21% of pediatric aneurysm cases, adjacent to other presenting features such as headache and loss of consciousness, with computed tomography revealing SAH in the majority of patients [9].

Diagnostic delay and emergency decisions

Diagnostic delay is one of the main lessons from this case. This child was missed because his earliest symptoms looked like a common pediatric illness. A week of headache and vomiting can be attributed to gastroenteritis or sinusitis, particularly when there is no fever, no focal deficit, and the child remains interactive. In hindsight, the key missed feature was the pattern. His symptoms were persistent, progressive, and not responding to initial treatment. That combination should prompt clinicians to reconsider a benign diagnosis and lower the threshold for neuroimaging.

This case also shows why low probability conditions still matter when the clinical course changes. Delayed intracranial hemorrhage after apparently minor or remote trauma is uncommon, but it does occur, and it is most likely to be missed when the initial presentation appears reassuring [10]. Even though the road traffic accident was one month earlier and he remained asymptomatic afterward, the persistence of headache with repeated vomiting should have triggered concern rather than reassurance. The child’s dramatic features appeared later, which is exactly why pediatric neurovascular events are often missed. Delays are frequently driven by low suspicion and delayed imaging after the patient reaches medical care [11]. Emergency literature similarly emphasizes that children may not look overtly neurologically unwell early on, leading to a prolonged time to definitive imaging when the symptoms are nonspecific [12]. Reviews further highlight limited provider familiarity and a lack of standardized pediatric recognition strategies, reinforcing a practical takeaway from this case. When a headache is progressive or atypical, or accompanied by neck stiffness or seizure, imaging should occur early, even if the initial bedside examination seems reassuring [13].

Contribution to the literature and future implications

This case highlights a high-risk diagnostic scenario in pediatric aneurysmal SAH. Clinically preserved neurological status can coexist with clinically significant hemorrhage and hydrocephalus, increasing the risk of diagnostic anchoring on benign conditions such as sinusitis or gastritis and delaying definitive evaluation [14]. In large cohorts of aneurysmal SAH, normal mental status at first medical contact has been independently associated with initial misdiagnosis, and misdiagnosis among patients initially presenting in good condition has been linked to worse downstream outcomes. This supports a deliberately low threshold for urgent neuroimaging and vascular evaluation when headache is progressive or atypical, or accompanied by seizure or meningeal signs [14,15].

From a pediatric neurovascular perspective, reporting this case adds value because pediatric aneurysms are rare and differ from adult disease in distribution and natural history, with series and reviews emphasizing frequent internal carotid involvement and a relatively higher representation of posterior circulation aneurysms in children compared with adults [16,17]. Treatment data also support individualized decision-making. A meta-analysis of pediatric aneurysm outcomes shows comparable rates of favorable clinical results after endovascular and surgical management across ruptured and unruptured lesions, while highlighting the need for stronger comparative and long-term data to refine pediatric-specific management strategies [18].

Long-term follow-up remains important even after successful treatment, as recurrence and de novo aneurysm formation have been documented in pediatric series, supporting structured longitudinal surveillance protocols [19].

## Conclusions

This case emphasizes how difficult it is to diagnose pediatric aneurysmal subarachnoid hemorrhage because nonspecific symptoms can initially mimic benign conditions and delay diagnosis. Our patient had a week of worsening headache and vomiting, followed by imaging-confirmed diffuse subarachnoid hemorrhage with intraventricular extension, classified as Hunt and Hess grade II, even though the Glasgow Coma Scale was preserved and there were no focal neurological deficits. This highlights the possibility of clinically significant bleeding even in cases where a bedside examination appears reassuring.

As evidenced by the patient's successful neurological recovery following timely stabilization, transfer to a specialized neurovascular center, and definitive management, early neuroimaging and coordinated referral are essential for children with ongoing or evolving neurological symptoms. In order to help avoid delayed diagnosis and lower potential morbidity, it is important to maintain a low threshold for neuroimaging in similar pediatric presentations, even though this is only one case.
